# SARS-CoV-2 drug resistance and therapeutic approaches

**DOI:** 10.1016/j.heliyon.2025.e41980

**Published:** 2025-01-15

**Authors:** Sania Batool, Santosh Chokkakula, Ju Hwan Jeong, Yun Hee Baek, Min-Suk Song

**Affiliations:** Department of Microbiology, Chungbuk National University, College of Medicine and Medical Research Institute, Cheongju 28644, Chungbuk, Republic of Korea

**Keywords:** Antiviral drugs, SARS-CoV-2, Drug resistance, Resistance mechanism, Therapeutic strategies

## Abstract

In light of the transition of COVID-19 from a pandemic to an endemic phase, there is still a dire need to address challenges associated with drug resistance, particularly among immunocompromised and high-risk populations. This review explores the current state of research on SARS-CoV-2 drug resistance and underscores the ongoing need for effective therapeutic strategies. It critically evaluates existing knowledge on resistance mechanisms and therapeutic options, aiming to consolidate information and highlight areas for future research. By examining the complex interactions between the virus and its host, the review advocates for a multifaceted approach, including combination therapies, targeted drug development, and continuous surveillance of viral mutations. It also emphasizes the impact of evolving viral variants on antiviral efficacy and suggests adaptive treatment protocols. This review aims to enhance our understanding of SARS-CoV-2 drug resistance and contribute to more effective management of COVID-19 through a discussion of promising strategies such as drug repurposing and combination therapies.

## 1 Introduction

Severe Acute Respiratory Syndrome Coronavirus 2, or SARS-CoV-2, is a novel coronavirus that emerged in late 2019 and caused the COVID-19 pandemic as declared by the World Health Organization in March 2020 [1]. By late 2020, scientists had made significant progress in the development of COVID-19 vaccines, intending to protect as many people as possible and to achieve herd immunity. However, the virus evolved, resulting in new variants such as Alpha, Beta, Gamma, Delta, and Omicron with varying degrees of transmissibility and potential resistance to immunity. These variants influenced the trajectory of the pandemic. These new variants not only initiated waves of infections but also helped people develop hybrid immunity, which provided some protection. As the virus evolved, it began to behave more like a normal seasonal virus, rather than causing severe outbreaks. By 2022, discussions had begun about the virus becoming less dangerous. For example, a newer variant, Omicron, spread more slowly in the lungs and caused less severe illness than older variants such as Delta [2]. This evolution made COVID-19 appear more like a regular illness, similar to the flu and led to the belief that it would become easier to manage with time. By May 4, 2023, amidst declining death and hospitalization rates, the WHO Director-General announced a shift towards long-term pandemic management for COVID-19 following the fifteenth report of the International Health Regulations (2005) Emergency Committee [3]. While the pandemic status has changed, ongoing research remains critical, particularly for vulnerable populations such as immunocompromised people and those at high risk. Immunocompromised individuals, such as those undergoing cancer treatment, organ transplant recipients, or those with untreated HIV often experience prolonged viral replication. It provides the virus with more opportunities to mutate and become resistant to antiviral treatments. Long-term infections, frequent use of antiviral drugs, and higher viral loads are all risk factors for mutations and resistance.

It was first identified in the city of Wuhan, the capital of China’s Hubei Province, investigations indicated a probable zoonotic origin from bats, with transmission to humans via an intermediate host [4,5]. It shares the family with two other closely related coronaviruses; including MERS-CoV, and SARS-CoV, that have previously caused significant outbreaks in 2012 and 2002–2003, respectively [6,7]. COVID-19 manifests with a spectrum of symptoms ranging from mild to severe, encompassing fever, cough, respiratory distress, anosmia (partial or full loss of smell), dysgeusia (distorted sense of taste), and, in most severe cases, Acute Respiratory Distress Syndrome (ARDS) and mortality.

SARS-CoV-2, classified within the Betacoronavirus genus, possesses a single-stranded RNA genome. Among its repertoire of structural and non-structural proteins encoded by this genome, the spike (S) protein plays a pivotal role in viral entry into host cells. SARS-CoV-2 has undergone mutations over time, leading to the emergence of various variants. Due to their increased transmissibility and potential to affect therapeutic effectiveness, some of these variants have raised considerable concern.

Antiviral drugs have played a crucial role in the management of the COVID-19 pandemic, offering valuable insights into potential treatments for viral outbreaks. While not a definitive cure, these drugs contribute significantly to symptom alleviation, severity reduction, and potentially expedited recovery. Their significance is particularly pronounced in cases of moderate to severe disease. Early administration of antiviral drugs can effectively decrease viral load in infected individuals, thereby diminishing their infectiousness and impeding viral transmission, thus breaking the chain of spread. Antiviral drugs offer an extra layer of defense for high-risk groups that are more vulnerable to severe COVID-19 outcomes, such as the elderly and immunocompromised patients—antiviral drugs aid in managing the disease in this highly susceptible population. Timely intervention with these drugs has demonstrated a notable decrease in hospitalization rates and mortality, pivotal in preventing healthcare system strain and preserving lives.

The emergence of SARS-CoV-2 variants has significantly altered the efficacy of certain treatments and vaccines. Variants such as Alpha (B.1.1.7), while more transmissible, had little effect on early drug resistance. Delta (B.1.617.2) variant, first identified in India in mid-2021, demonstrated increased transmissibility and severity, as well as some resistance to monoclonal antibodies due to mutations in the spike protein, particularly the L452R and T478K which contributed to this altered drug resistance profile [8]. Omicron (B.1.1.529), identified in South Africa in late-2021, and its subvariants (e.g., BA.1, BA.2, BA.4, BA.5) caused numerous mutations, reducing the efficacy of monoclonal antibodies and potentially affecting some antiviral drugs [9]. Treatment strategies became more complicated due to the rapid evolution of sublineages such as BA.1, BA.2, and BA.5, as each sublineage exhibited distinct resistance patterns [10,11,12]. Beta (B.1.351) and Gamma (P.1) were also less susceptible to monoclonal antibodies which is probably because of the mutations at the 417, 484, and 501 amino acid positions in the spike RBD of both variants [12]. As of June 28, 2024, the only currently circulating Variant of Interest (VOI) is JN.1 after WHO excluded BA.2.86 from the list [13]. JN.1 is a sublineage of the Omicron variant that is grabbing more attention recently due to its unique mutations, especially the L455S mutation in the receptor-binding motif (RBM) of the RBD [14]. Approved antiviral drugs continue to be effective against BA.2.86 and JN.1. Nevertheless, developing antiviral drugs requires urgent attention because these variants are resistant to many monoclonal antibodies. Although the renewed monovalent XBB.1.5 vaccine protects against JN.1, induced neutralizing antibody titers are significantly lower than in the wild-type [14]. In contrast, the XBB.1.5 trivalent mRNA vaccine maintains high neutralizing titers, even surpassing those seen in XBB breakthrough infections [14].

Antiviral drugs with various modes of action can be used for variant-specific treatment and offer tailored options for treatment when the efficacy of other interventions is compromised. However, the management of the pandemic is significantly hampered by the development of drug resistance. SARS-CoV-2 mutations can render antiviral drugs ineffective or only partially effective, often in response to the selective pressure exerted by these drugs. Consequently, the efficiency of antiviral treatments diminishes as drug-resistant strains proliferate, posing challenges in symptom management and viral load reduction for COVID-19 patients reliant on these drugs. Continued infection with drug-resistant variants may exacerbate case numbers, while variants bearing drug-resistant mutations may concurrently harbor mutations rendering vaccines less effective. This dual challenge complicates efforts to curb virus spread and attain herd immunity.

Monoclonal antibodies represent a vital component of the immune system’s arsenal against deadly pathogens such as viruses. In the context of the SARS-CoV-2, several monoclonal antibodies have been developed and approved for emergency use COVID-19 treatment. These antibodies are developed to specifically target and impede the virus’s ability to infect human cells. Notably, several monoclonal antibodies, such as Bamlanivimab, Etesevimab, Casirivimab, Imdevimab, Tixagevimab, Cilgavimab, Sotrovimab, Regdanvimab, and Bebtelovimab, have received authorization from regulatory bodies like the Food and Drug Administration (FDA) and the European Medicines Agency (EMA). However, some of these authorizations have been subsequently revoked or restricted after a brief period.

The efficacy of monoclonal antibody treatments becomes compromised as the virus evolves, acquiring mutations within the epitopes targeted by these antibodies. This phenomenon has been particularly evident with certain variants, notably those within the omicron lineage. Liu et al. first observed the potential emergence of neutralization-resistant variants when monoclonal antibodies were used in laboratory conditions (*in vitro*) [15]. Later, Fenaux et al. reported a case of an immunocompromised patient who displayed prolonged and high viral loads for 51 days despite Bamlanivimab and Etesevimab therapy, with sequencing revealing mutations at targeted monoclonal antibody positions, suggesting resistance to the treatment [16]. In early 2022, the FDA imposed restrictions on specific monoclonal antibody treatments following emerging data indicating their reduced effectiveness against the prevailing omicron variant of COVID-19 [17]. Numerous monoclonal antibody treatments have demonstrated ineffectiveness against Omicron and its highly transmissible subvariants. Accordingly, the FDA revoked the emergency use authorization for the monoclonal antibody therapy EVUSHELD in January 2023 due to its inability to confer protection against the latest and most prevalent strains of COVID-19 in the US. For the same reason, the FDA revoked bebtelovimab's approval in late 2022 [18].

This review paper aims to comprehend the challenges posed by drug resistance in the COVID-19 pandemic by digging deep into the diverse mechanisms underlying SARS-CoV-2 resistance. Furthermore, it would advance scientific understanding and assist in the development of effective therapeutic plans. Despite the transition away from pandemic status, the importance of ongoing research in mitigating the impact of COVID-19 on vulnerable populations remains of great importance.

Our review paper distinguishes itself from the existing literature by providing a comprehensive and integrated evaluation of both drug resistance and therapeutic strategies against SARS-CoV-2. Although most of the reviews have usually focused on either drug resistance or specific classes of therapeutic approaches, our paper combines these aspects to provide a comprehensive understanding of their dynamics. Furthermore, our review article includes the most recent studies and data, providing updated insights into emerging resistance patterns and novel treatment options that were not covered altogether in previous reviews. This review serves as a one-stop resource for researchers, offering a broad range of aspects and a comprehensive summary of SARS-CoV-2. We identify critical gaps in current knowledge and propose innovative solutions by taking an interdisciplinary approach that includes virology, pharmacology, and clinical perspectives, thereby advancing the field in a unique and valuable direction.

## 2 SARS-CoV-2 drug resistance mechanism

### 2.1 Overview of viral replication and mutation rate

RNA viruses exhibit a notably high mutation rate owing to the inherent instability of their genetic material, RNA, in comparison to DNA. This heightened propensity for mutation stems from several factors, including a lack of proofreading mechanisms, the presence of quasispecies, and the phenomenon of error catastrophe. These mutations, particularly in target proteins, can confer resistance to antiviral drugs, posing challenges to treatment efficacy. Unlike DNA polymerases, RdRp enzymes lack proofreading capabilities, thereby permitting the perpetuation of replication errors. However, it is noteworthy that viruses within the coronavirus family possess RdRp-independent proofreading activity, mitigating their mutation rates to some extent [19].

The viral replication process of SARS-CoV-2 involves specific steps within host cells and exhibits a moderate yet significant mutation rate. It is critical to uncover how SARS-CoV-2 interacts with host factors during its life cycle to fully comprehend the pathogenic mechanisms that underlie the viral infection and to devise effective prophylactic measures and antiviral therapies. SARS-CoV-2 viral replication is initiated by infecting human respiratory epithelial cells, where the virus adheres to surface receptors of the host cell, facilitated by the spike protein present on the viral envelope. The spike protein binds to the human angiotensin-converting enzyme 2 (hACE2) via the receptor-binding domain (RBD), inducing major conformational changes necessary for infection [20,21]. Following viral attachment, the viral envelope either fuses with the host cell membrane, allowing the entry of the viral ribonucleoprotein complex into the cytoplasm [22], or undergoes endocytosis before fusion [21]. Upon entry into the host cell, the viral RNA is released and serves as a template for transcription and translation. Two open reading frames, ORF1a and ORF1b, encoded within the viral RNA, are translated into viral proteins, pp1a, and pp1ab, respectively, by the host cell’s ribosomes. This process yields the replicase-transcriptase complex (RTC), crucial for viral RNA genome replication, occurring within protective double-membrane vesicles (DMVs) in the host cell's cytoplasm [21]. Viral RNA polymerase is responsible for catalyzing the replication of the RNA genome, which produces subgenomic RNA (which is utilized to produce viral proteins), as well as genomic RNA (which is utilized to create new viral particles). Subsequently, the full-length genomic RNA generates the subgenomic RNAs which act as templates, facilitating the synthesis of viral structure and accessory proteins. These subgenomic RNAs are translated into proteins by the host cell’s machinery, and viral proteins assemble into new virions. Structural proteins including spike(S), envelope(E), membrane(M), and nucleocapsid(N) proteins aid in budding at the endoplasmic reticulum (ER) to the Golgi compartment. Virions are then released from the infected cells into the extracellular space via exocytosis or by insertion into lysosomes, subsequently fusing with the cell membrane [21]. This viral replication cycle keeps going on by releasing virions infecting the other host cells.

“The mutation rate is a function of replication fidelity” [23]. Mutations occur every time a virus replicates inside a cell. Generally, the mutation rates for the various positive sense ssRNA viruses that have been studied range from 10^6 to 10^3 and are expressed as nucleotide substitution per-site per-cell infection (s/n/c) [24]. SARS-CoV-2 exhibits an unstable mutation rate, initially characterized by low mutation frequency. However, as the pandemic progressed, the mutation rate escalated. Different segments of the virus evolve at varying rates; while certain components such as nsp11, nsp7, nsp10, nsp8, and nsp16 undergo gradual changes and remain relatively stable, others like N, nsp3, S, nsp4, nsp12, and M proteins mutate at a faster rate [25]. The mutation rate also varies among viral lineages and across different regions of the virus genome, with mutations accumulating more rapidly in some regions than in others. Despite its RNA nature, SARS-CoV-2 displays a lower mutation rate compared to other RNA viruses, notably HIV [26] and influenza virus (H3N2) [27]. The majority of the mutations in the SARS-CoV-2 genome are point mutations, altering a single nucleotide in the viral RNA. The spike protein, which is the target of numerous vaccinations and therapies, is one of the many locations in the genome where point mutations can arise. Early in the pandemic, under neutral genetic drift conditions, the mutation rate was estimated to be approximately 1 × 10^3 substitutions per base (per year) [28], implying an average occurrence of one mutation in the viral genome every 1000 years per nucleotide. Further studies have stated a slight increase in mutation rates, such as 1 × 10^5 to 1 × 10^4 substitution per base per transmission event [29]. In late December 2019, researchers analyzed 32 genome sequences of SARS-CoV-2 and stated an estimated mean evolutionary rate ranging from 1.7926 × 10^3 to 1.8266 × 10^3 substitutions per site per year [30]. Between February and April 2020, the Afr-SARS-CoV-2 evolved at a rate of 4.133 × 10^4 substitutions per site per year [31], offering insights into the virus’s evolving pace. Based on the findings of Amicone et al. the estimated mean rate of spontaneous mutation in two lineages of SARS-CoV-2 (one with the original spike protein and the other with D614G mutation) is 1.3 × 10^6 ± 0.2 × 10^6 per base per infection cycle [23]. These findings contribute significantly to our understanding of the dynamic evolution of the virus and its implications for transmission and adaptation.

### 2.2 Overview of available antiviral drug

While numerous antiviral drugs have undergone rigorous testing against SARS-CoV-2, only a few have garnered approval for clinical application (Table 1).

(i) Paxlovid (Nirmatrelvir + Ritonavir): Developed by Pfizer, Paxlovid comprises two active ingredients; nirmatrelvir and ritonavir [32]. The FDA has approved Paxlovid as the first oral antiviral medication and the fourth drug to combat COVID-19 [33]. Nirmatrelvir or PF-07321332, serves as a protease inhibitor that blocks the viral replication at the proteolysis stage [32] by acting on Mpro (also known as 3CLpro or nsp5). Ritonavir is co-formulated with nirmatrelvir to act as a ‘booster’, elevating nirmatrelvir levels and efficacy by inhibiting cytochrome P 450 (CYP) 3A, an enzyme responsible for its breakdown. The approval of Paxlovid, which was supported by the EPIC-HR clinical trial [34], represents a significant step forward in the fight against COVID-19. Paxlovid showed an 89 % reduction in the risk of COVID-19-related hospitalization or death within 28 days when administered within five days of symptom onset and without prior COVID-19 therapeutic monoclonal antibody treatment, according to a randomized, double-blind, placebo-controlled trial focusing on non-hospitalized symptomatic adults. Notably, these benefits were observed even among patients with prior immunity [34].
(ii) Remdesivir: Remdesivir is a broad-spectrum antiviral drug with potential efficacy against various RNA viruses [35]. In its active form, remdesivir functions as an adenosine analog, inhibiting the viral RNA-dependent RNA-polymerase (RdRp) in SARS-CoV-2 and other coronaviruses. Once integrated into the RNA chain by RdRp, remdesivir facilitates the addition of three more nucleotides before terminating RNA synthesis. The effectiveness of remdesivir was initially demonstrated by Wang et al. [36] in *in vitro* experiment, with an EC50 value of 0.77μM in Vero E6 cells. Subsequently, in a double-blind, randomized, placebo-controlled trial, hospitalized SARS-CoV-2 patients receiving a 10-day course of remdesivir treatment exhibited notable benefits [37]. In a comparison between the remdesivir and placebo arm, the former had a shorter recovery period, lower mortality, and improved key secondary endpoints as per the study [37]. Recognizing its potential, the Food and Drug Administration (FDA) issued an Emergency Use Authorization (EUA) on May 1, 2020, permitting the use of remdesivir for the treatment of adults and children hospitalized with suspected or confirmed COVID-19 [38].
(iii) Molnupiravir: Molnupiravir, marketed under the tradename Lagevrio, was granted EUA by the FDA on December 23, 2021, for the treatment of mild-to-moderate COVID-19 disease in adults who have tested positive for SARS-CoV-2 and are at high risk of developing severe Covid-19. Later, on February 1, 2023, the FDA revised the EUA for Lagevrio and eliminated the need for positive SARS-CoV-2 test results before prescribing.

**Table 1:** Approved Antiviral Drug against SARS-CoV-2 and their Mechanisms.

Sr. No.	Drug	Class	Mechanism of Action	Brand Name	Administration Route	Approval Agency	Approval Year
**1**	Nirmatrelvir + Ritonavir	3CL protease inhibitor + CYP3A inhibitor	Inhibits the coronavirus's viral replication cycle	Paxlovid	Oral	FDA	2023
**2**	Remdesivir	Nucleoside Analog	Inhibits the RNA-dependent RNA polymerase (RdRp) of coronaviruses	Veklury	Intravenous	FDA	2020
**3**	Molnupiravir	Ribonucleoside Analog	Inhibits SARS-CoV-2 replication by viral mutagenesis	Lagevrio	Oral	UK (MHRA)	2021
**4**	Ensitrelvir	3CL protease inhibitor	Suppresses the SARS-CoV-2 replication by selectively inhibiting the 3CL protease	Xocova	Oral	Japan (MHLW)	2022
**5**	Favipiravir	Pyrazine	Inhibits the viral replication by targeting the RdRp	Avigan, Coronavir (Russia)	Oral	India (DCGI), Russia	2020
**6**	Azvudine	Nucleoside Analog	Acts as a reverse transcriptase inhibitor	Two words combined; 双新艾克 (literally "dual new AIDS inhibitor") for HIV use and 捷倍安 (literally "fast extra safe"	Oral	China (NMPA)	2022
**7**	Simnotrelvir + Ritonavir	3CL protease inhibitor + CYP3A inhibitor	Targets 3CL protease and inhibits the SARS-CoV-2 viral replication	Xiannuoxin	Oral	China (NMPA)	2023
**8**	Deuremidevir/VV116	Nucleoside Analog	Inhibits the RdRp of SARS-CoV-2 and exerts antiviral effects	Min De Wei (民得维)	Oral	Uzbekistan, China (NMPA)	2021, 2023
**9**	Leritrelvir	Nucleoside Analog	Selective inhibitor of the main protease of the coronavirus	Le Rui Ling (乐睿灵)	Oral	China (NMPA)	2023

**FDA**—Food and Drug Administration, **MHRA**—Medicines and Healthcare Products Regulatory Agency, **MHLW**—Ministry of Health, Labour and Welfare**, DCGI**—Drug Controller General of India, **NMPA**— National Medical Products Administration.

Molnupiravir is rapidly transformed into an active form in the bloodstream with the aid of host kinases. This active form accumulates mutations during viral replication by competing with the viral RdRp, thereby exerting its antiviral effect. Preclinical studies have demonstrated its broad-spectrum antiviral efficacy against coronaviruses, including SARS-CoV-2, exhibiting a high resistance barrier [39,40]. Molnupiravir treatment has been shown to inhibit virus replication and prevent transmission in mice and ferrets models [41,42,43]. Its safety profile has been verified by phase 1 trials involving healthy volunteers [44].

Molnupiravir showed antiviral efficacy against SARS-CoV-2 in a phase 2a clinical trial [45], as evidenced by lower infectious virus isolation, faster viral RNA clearance, and a higher proportion of participants clearing the virus when compared to placebo. Safety analyses supported ongoing clinical development, indicating that molnupiravir was well tolerated with no significant adverse events, implying that it has the potential as an oral agent to reduce viral replication and progression of COVID-19 in the early stages of the disease.

(iv) Ensitrelvir: Ensitrelvir (S-217622) represents a significant advancement in combating SARS-CoV-2, having garnered an EUA in Japan based on its notable performance in a phase 2/3 clinical trial [46]. Unlike Nirmatrelvir, Ensitrelvir targets the 3CLpro without requiring a pharmaceutical booster for its antiviral activity, presenting a notable advantage. Its development involved comprehensive evaluation and was introduced as the “first oral noncovalent, nonpeptidic clinical candidate” targeting the virus’s 3CL protease [47]. The discovery involved virtual screening, followed by biological screening of an internal compound repository, and structure-based drug design to refine the initial hit compound [47]. Studies by Lin et al. elucidated the Mpro-ensitrelvir complex, revealing ensitrelvir utilization of stable hydrogen bonding in the substrate-binding pocket to interact with specific sites on Mpro, a mechanism distinct from that of nirmatrelvir [48]. Given the essential role of N-terminus residues in maintaining substrate pocket stability within the Mpro homodimer, ensitrelvir demonstrates stronger *in vitro* inhibition of Mpro compared to nirmatrelvir. However, despite promising outcomes from clinical trials, there remains a dearth of data on the effectiveness and safety of Ensitrelvir in clinical practice.
(v) Favipiravir: Favipiravir (T-705), akin to remdesivir, functions by inhibiting the RNA-dependent RNA polymerase enzyme, showcasing efficacy against several RNA viruses such as influenza. Initially approved in Japan for influenza treatment, Favipiravir has garnered attention for treating SARS-CoV-2 infections in multiple Asian countries [49], halting virus proliferation and dissemination within the body. Laboratory studies have demonstrated Favipiravir's inhibition of COVID-19 activity [50], suggesting its potential to impede various aspects of the virus's replication process, including RNA synthesis, thereby inducing unfavorable mutations and halting the replication chain [51]. Recognized as a potent COVID-19 treatment, particularly when administered early in the disease course [52], Favipiravir's ability to curtail virus replication may diminish transmission risk and mitigate disease progression to severe stages [53]. Its oral administration further enhances accessibility for both hospitalized and non-hospitalized patients with mild to moderate illness [54].

Favipiravir was first used in Wuhan during the beginning of the COVID-19 pandemic. Since then, it has been recognized and approved for use in emergencies across the globe, including Saudi Arabia, UAE, Italy, Ukraine, Japan, Russia, Uzbekistan, Kazakhstan, Egypt. Bangladesh, Turkey, and Moldova. In June 2020, the Drug Controller General of India (DCGI) approved the treatment of mild and moderate COVID-19 infections [52].

(vi) Azvudine: Azvudine, renowned as a reverse transcriptase inhibitor, initially developed for Hepatitis C treatment [55], has drawn attention for potential applications against viral diseases including COVID-19 [56] and AIDS [57]. Ongoing evaluation and its potential therapeutic relevance in managing viral infections have led to conditional approval in China for COVID-19 treatment [58].

Researchers conducted a study comparing the efficacy of oral Azvudine and Paxlovid in treating symptomatic adults hospitalized with mild to moderate COVID-19 during the January–February 2023 outbreak, focusing on the omicron subvariants BF.7 and BA.5.2 [59]. The study revealed that a 7-day course of oral azvudine exhibited a robust safety profile and was non-inferior to Paxlovid in reducing hospitalization time, mortality rates, and time required for sustained clinical recovery. Despite a longer median time to sustained clinical recovery in this trial compared to another, likely due to the initiation of medication beyond the first five days of infection, azvudine demonstrated efficacy. Improvements in clinical recovery were associated with vaccination, consistent with recent Paxlovid studies.

(vii) Xiannuoxin: (Simnotrelvir/ritonavir): The Shanghai Institute of Materia Medica, the Wuhan Institute of Virology, and Simcere Pharmaceutical Group Limited collaborated to develop the innovative oral anti-SARS-CoV-2 drug Xiannuoxin [60]. The National Medical Products Administration of China has granted conditional approval for the drug’s marketing in 2023 [58]. This novel drug targets the 3CL protease, crucial for SARS-CoV-2 replication, making the first anti-SARS-CoV-2 drug with intellectual property rights in China. Xiannuoxin combines low-dose ritonavir and Simnotrelvir, exhibiting strong, broad-spectrum anti-SARS-CoV-2 activity in preclinical studies. Moreover, it has demonstrated safety and efficacy in multi-center clinical trials involving 1208 adult patients with mild to moderate COVID-19 infection [61].
  (viii) VV116 (Deuremidevir): The National Medical Products Administration of China has also granted conditional approval for the marketing of VV116, another excellent oral anti-SARS-CoV-2 drug [62]. VV116, a nucleoside analog antiviral drug jointly developed by the Wuhan Institute of Virology, the Shanghai Institute of Materia Medica, and several other organizations, is protected by intellectual property rights in China. It exerts its inhibitory effects on virus replication by non-covalently binding to the active center of the RNA-dependent RNA polymerase of SARS-CoV-2, as demonstrated in preclinical studies against both the original Covid-19 strain and mutant strains such as Omicron [63]. Phase III clinical trials led by academician Li Lanjuan revealed that VV116 not only decreased the time to sustained resolution of clinical symptoms but also exhibited superior virological indicators compared to the placebo group, rendering it a promising addition to COVID-19 treatment options in China [64].
(ix) Leritrelvir:

RAY1216, also known as leritrelvir, an oral antiviral drug developed by Guangdong Zhongsheng Pharmaceutical, received conditional market approval from the National Medical Products Administration China in March 2023 for COVID-19 treatment [65]. This approval allows its use in adults experiencing mild to moderate viral symptoms under medical supervision. As an oral 3CLpro inhibitor, leritrelvir has demonstrated strong anti-SARS-CoV-2 efficacy in preclinical studies and exhibited favorable safety profiles across various dosages in a phase 1 clinical study. In a randomized, double-blind clinical trial involving 60 adults with COVID-19 during the Omicron variant prevalence period, both RAY1216 alone and in combination with ritonavir effectively reduced SARS-CoV-2 replication, significantly accelerating viral shedding compared to the placebo group within 5 days of positive nucleic acid results [66]. In a recent Phase 3 trial, leritrelvir without ritonavir booster significantly shortened recovery times for mild-to-moderate COVID-19 symptoms compared to placebo [67]. This drug’s favorable safety profile and demonstrated effectiveness in reducing viral load allay concerns regarding potential drug interactions. Its notable effectiveness against the Omicron variant indicates a promising future in treating SARS-CoV-2 infections.

### 2.3 Key targets of antiviral drugs

For a better understanding of the drug resistance mechanism of SARS-CoV-2, it is essential to elucidate the key targets of the antiviral drugs (Table 2). The life cycle of SARS-CoV-2 comprises important stages; primarily (1) Entry/Invasion 2) Replication 3) Genome-assembly 4) Release offering potential opportunities for developing treatments for COVID-19. These critical checkpoints serve as targets for therapeutic interventions, categorized into three main groups:

**Table 2:** Overview of SARS-CoV-2 drug targets and resistance mechanism.

Drug Targets	Resistance Mechanism
**Spike Protein**	Genetic mutations in the S protein influence the virus’s interaction with the host and change its susceptibility to neutralization by antibodies
**PLpro**	Genetic mutations in the PLpro region alter the enzyme’s structure or function, which may affect the virus’s ability to evade host immune responses or resist antiviral drugs that target PLpro
**Mpro**	Genetic mutations in the Mpro coding region alter the structure or function of the enzyme, which can impact host-cell interaction, viral replication efficiency, and susceptibility to antivirals that target Mpro
**RdRp**	Genetic mutations, particularly in the S759DD active motif of the nsp12-RdRp region, result in varying degrees of reduced susceptibility across multiple viral lineages

#### 2.3.1 Virus-based targets

(i) PLpro: The papain-like protease (PLpro), a key enzyme within the SARS-CoV-2 replication machinery, cleaves viral polyproteins and host cell proteins, making it the primary antiviral target. Inhibiting PLpro activity could impede viral replication and dissemination within the host. Targeting peripheral PLpro sites could be a promising antiviral strategy [68].
(ii) Spike Protein: The SARS-CoV-2 relies on the Spike protein (S protein) for host cell entry, making it a significant therapeutic target. The S protein, cleaved by a furin-like protease, consists of two subunits—S1 and S2—where the former binds to host cell receptors, and the latter mediates viral and host cell membrane fusion, crucial for infection. This division of labor within the S protein is essential for the virus to enter and infect host cells. The receptor binding domain (RBD), located within the S1 subunit, facilitates the interaction between S protein and the ACE2 receptor, enabling viral entry into host cells [69]. Consequently, inhibiting the RBS-ACE2 interaction emerges as a crucial target for potential treatments.
(iii) 3CLpro/Mpro: S protein of SARS-CoV-2 is more prone to mutations, with notable increases observed in current SARS-CoV-2 variants, potentially compromising vaccine effectiveness [70]. As a result, researchers are exploring alternative antiviral targets, with enzymes emerging as promising candidates due to the rarity of mutations affecting their active sites. The 3CLpro (Main protease) stands out as a favorable antiviral drug target, playing a crucial role in the viral life cycle by cleaving large polyproteins into functional viral proteins necessary for replication. Inhibiting 3CLpro activity can disrupt viral replication [71] and may prove to be an effective strategy to combat SARS-CoV-2.
(iv) RdRp: RNA-dependent RNA-polymerase (also known as nsp12) serves as a predominant antiviral drug target for SARS-CoV-2 [72]. RNA-dependent RNA polymerases facilitate the replication of RNA-based viruses. Inhibiting RdRp has been effective in treating various viral infections, including dengue, hepatitis C, Zika, chikungunya, influenza, and bovine viral diarrhea virus. Although RdRp shares structural elements such as motifs and domains with DNA and RNA polymerases, each RdRp inhibitor may interact differently with its binding pocket [73]. It implies that each RdRp inhibitor may share structural characteristics or binding conformations contributing to similar inhibitory effects observed across the RNA virus family.

#### 2.3.2 Host-based targets

(i) ACE2: The interaction between Angiotensin-Converting Enzyme (ACE2) and SARS-CoV-2 plays a pivotal role in the viral infection process. As previously mentioned, the S protein of SARS-CoV-2 binds to the ACE2 receptor on the surface of human cells, particularly those within the respiratory system, facilitating viral entry and initiation of infection. The interaction is implicated in COVID-19 symptoms and lung inflammation by downregulating ACE2 expression and inducing an inflammatory response. Initially, researchers suggested some ACE2 inhibitors based on In silico studies [74,75,76,77] but attention shifted to the SARS-CoV-2 S protein: ACE2 complex upon realization of the virus’s binding mechanism. Nitrofurantoin, favipiravir, eriodictyol, cepharanthine, ergoloid, hydroxychloroquine, thymoquinone, raspberry-ketone, hypericin, isoniazid pyruvate, and hydroxychloroquine emerged as potent inhibitors of SARS-CoV-2 S protein-ACE2 complex [78,79]. Furthermore, the SARS-CoV-2 S-protein receptor binding domain (S-RBD) shares a high similarity with SARS-CoV strains found in bats, humans, and palm civet cats, with ACE2 exhibiting approximately tenfold higher affinity for SARS-CoV-2 S-RBD compared to SARS-CoV RBD. It suggests ACE2’s pivotal role as the primary receptor mediating virus attachment to the host cell membrane. Consequently, researchers have directed their focus toward inhibitors targeting the SARS-CoV-2-RBD-ACE2 interaction complex, reporting inhibitors from both synthetic [80,81,82,83] and natural sources [84,85,86,87,88,89,90,91].
(ii) TMPRSS2: Pharmacological inhibition of type-II transmembrane serine proteases (TTSPs), such as Transmembrane Protease, Serine 2 (TMPRSS2) is a promising strategy for inhibiting SARS-CoV-2 entry into host cells. TMPRSS2 plays an important role in the virus’s life cycle by cleaving the viral spike proteins, initiating conformational changes that expose the fusion peptides, and facilitating the fusion between viral and host cell membranes. Researchers are making efforts to identify the inhibitors targeting TMPRSS2, with Shapira et al., 2022 [92] developing a potent inhibitor, N-0385, demonstrating efficacy against TMPRSS2-like proteases and various SARS-CoV-2 variants of concern (VOCs), including B.1.1.7, B.1.351, and Delta variant. Additionally, researchers have repurposed existing peptidyl inhibitors to identify robust TMPRSS2 inhibitors, such as MM3122, showing superior antiviral efficacy compared to other well-known drugs like Remdesivir [93].
(iii) Cathepsin L: Cathepsin L, a lysosomal enzyme that aids in the protein breakdown during immune responses to pathogens, has emerged as a potential target for drugs aiming to combat SARS-CoV-2. While CTSL's involvement in SARS-CoV-1 virus fusion with host cells under laboratory conditions has been documented [94], its role in SARS-CoV-2 infection remained speculative until highlighted by Zhao et al., 2021 [95]. The study highlighted the potential of CTSL as an important therapeutic target for SARS-CoV-2, revealing that viral infection amplifies CTSL expression and activity, facilitating viral entry through cleavage of SARS-CoV-2 proteins. Notably, they found that the well-known anti-influenza drug amantadine efficiently suppresses CTSL activity after SARS-CoV-2 pseudovirus infection, thereby averting infection in both *in vitro* and *in vivo* experiment models [95].
(iv) Furin: Furin is a proprotein convertase enzyme that plays a crucial role in cleaving viral spike proteins at the PRRAR-containing S1/S2 cleavage site. This cleavage event facilitates the activation of the S protein, enhancing the virus’s ability to enter host cells more efficiently. The importance of cleaving the spike protein was confirmed by Cheng et al. [96], who identified furin inhibitors, specifically CMK, and naphthofluorescein, capable of effectively blocking this cleavage process and subsequent syncytium formation, a crucial step in viral fusion with host cells. These inhibitors demonstrated reductions in virus production and cell damage, with CMK inhibiting virus entry, spike protein cleavage, and syncytium formation, while naphthofluorescein primarily targeted viral RNA transcription [96]. These findings suggest that furin inhibitors hold promise as valuable antiviral agents for both the prevention and treatment of SARS-CoV-2 infection.

### 2.4 Factors contributing to the emergence of drug resistance

In 2022, Antonio Vitiello wrote a letter to the editor in which he expressed his concern about SARS-CoV-2’s potential to develop antiviral drug resistance, highlighting the possibility that this could result in clinical treatment failures [97]. Several factors contribute to the emergence of resistance in SARS-CoV-2, including:

i) *High Replication Rate:* SARS-CoV-2, like other RNA viruses, replicates rapidly [98], generating numerous viral copies. Errors in the viral genome can occur during viral replication, leading to changes in the targeted antiviral drug proteins. Resistance may arise if a mutation renders the virus to avoid the inhibitory effects of a drug.
ii) *Drug Pressure:* Antiviral drugs exert selective pressure on the SARS-CoV-2 when prescribed, prompting rapid viral evolution in response to new selection pressures [99,100]. The drugs are developed to specifically target viral proteins or replication-related processes. While drug-sensitive viral strains are suppressed after treatment with drugs, some viral variants with mutations might persist and replicate, potentially leading to drug resistance.
iii) *Prolonged Exposure:* The duration of antiviral treatment can influence the development of drug resistance in SARS-CoV-2, as longer regimens provide more opportunities for the virus to encounter drug-induced selective pressure. Increased exposure increases the likelihood of viral replication and the acquisition of resistance mutations.
iv) *Incomplete Viral Suppression:* Incomplete antiviral treatment reduces existing infections and offers protection to uninfected target cells. Nevertheless, the new viruses that escape the treatment are still able to initiate new infections in the previously protected, uninfected cells, albeit at a slower rate [101]. This incomplete viral suppression allows some viral strains to survive, mutate, and become resistant to the drugs.
v) *Immune Escape:* The “immune escape” describes a virus’s capacity to elude the host’s defenses, including neutralizing antibodies. Once the virus manages to elude the immune system’s recognition and clearance mechanisms, it can continue to replicate and spread throughout the host. Increased viral loads may result from this [102].
vi) *Inappropriate Viral Use*: Inappropriate or inconsistent use of antiviral drugs, such as underdosing, inadequate combinations, unapproved use, self-medication, or not completing the full course of treatment, can promote the development of resistance. Underdoing or overdosing on Nirmatrelvir/Ritonavir may contribute to the selection of Nirmatrelvir-resistant SARS-CoV-2 strains, which is concerning given the history of HIV-1 resistance to protease inhibitors [103].
vii) *Combinations of Mutations:* Combinations of mutations in SARS-CoV-2 [104] can change the virus’s genetic makeup and how it reacts to antiviral drugs, which can lead to antiviral resistance by cumulative effects, multiple drug targets, resistance to drug combinations, and by creating complex resistance profiles. Multiple mutations can make antiviral therapy management more difficult.
viii) *Global Spread:* Through several interrelated mechanisms, the global spread of SARS-CoV-2 may indirectly contribute to the emergence of antiviral drug resistance. That includes diversity of variants, increased replication, transmission of resistant variants, selective advantage, complex resistance patterns, global mobility, and altered treatment efficacy.
ix) *Overuse:* Antiviral drug resistance can arise from the overuse of antiviral drugs in the treatment of SARS-CoV-2 through several mechanisms, such as; high levels of drug pressure due to overusing the drug, which in turn suppress the replication of drug-sensitive viral strains, but drug-resistant strains keep replicating [105].

### 2.5 Mechanism of drug resistance in viruses

Drug resistance in RNA viruses, including SARS-CoV-2, emerges through several intricate mechanisms that challenge efforts to control and eradicate viral infections. One key mechanism is latency. For example, viruses in the Herpesviridae family can enter a latent state within the host, allowing them to persist for a lifetime even with a functional immune system [106]. These dormant viruses can reactivate periodically, often without symptoms, making complete eradication difficult. In addition, certain viruses, like HIV, integrate a DNA copy of their RNA genome into the host cell’s DNA, becoming part of the host's genetic material and contributing to persistent infection [107].

Another factor contributing to resistance is the diversity of viral genotypes and serotypes [108]. While antiviral drugs and vaccines may be highly effective against specific strains, their protective benefits can be limited or ineffective against other strains.

RNA viruses also exhibit unique resistance mechanisms. For instance, the influenza virus, with its segmented genome, can undergo genetic reassortment when two strains co-infect a single cell [109]. This reassortment can create new virus variants, potentially combining characteristics of drug-sensitive and drug-resistant strains, resulting in highly contagious viruses that are difficult to control.

Viruses use random mutations as their primary mechanism to develop resistance to antiviral treatments and vaccines, aligning with Darwin’s theory of evolution where random changes lead to the survival of the fittest [110]. Rapidly mutating viruses generate numerous variants known as quasi-species, some of which, when subjected to antiviral pressure, result in a dominant resistant strain, demonstrating the selective survival of the fittest [108].

Viruses may also develop resistance by altering their replication cycle or modifying the drug target itself [111]. Additionally, increased expression of drug efflux pumps can expel drugs from infected cells, and some viruses may enhance drug metabolism or fail to activate prodrugs properly. For example, antiviral drugs like M2 channel blockers can lead to rapid, widespread resistance due to single mutations, limiting their clinical utility [112]. Conversely, drugs that mimic viral enzyme substrates often experience a slower development of resistance [113]. Initially, resistance may arise from mutations that disrupt drug binding without significantly affecting the virus’s catalytic activity. Over time, secondary mutations may impact viral fitness, and tertiary mutations can further adapt the virus, sometimes in ways unrelated to drug interaction sites.

Cross-resistance is another important phenomenon, where resistance to one drug can confer resistance to other drugs with similar mechanisms [114]. Compensatory mutations may also occur, helping the virus maintain its fitness despite the presence of resistance mutations [114].

#### 2.5.1 Impact of glycans on SARS-CoV-2 resistance mechanisms

*Glycans are crucial to SARS-CoV-2's resistance mechanisms, impacting both viral infectivity and immune evasion. The spike (S) protein and ACE2 receptor, which are both extensively glycosylated, illustrate how glycans can influence viral interactions and resistance. Specific N-glycans on the spike protein, such as those at positions N90 and N322, modulate the binding affinity for ACE2 and affect viral infectivity by altering the spike protein's conformation and stability* [115]*. Moreover, glycosylation can shield the virus from immune recognition, aiding in evasion of neutralizing antibodies and diminishing vaccine efficacy* [116]*. Inhibitors of glycosylation, such as mannosidase-I inhibitors, have demonstrated significant reductions in viral entry by disrupting the spike protein's glycosylation pattern, thereby highlighting the role of glycans in SARS-CoV-2's resistance and suggesting potential therapeutic interventions* [117]*. Understanding these glycan-mediated processes is essential for developing more effective vaccines and treatments against SARS-CoV-2 variants.*

Overall, resistance evolves through a dynamic and complex interplay of mutations and their interactions, underscoring the challenges in developing effective antiviral therapies. Resistant strains can cause treatment failures, necessitating changes in therapy and possibly worsening patient outcomes. The spread of resistant strains complicates public health efforts and raises the possibility of widespread resistance. As resistance reduces the efficacy of available drugs, new antiviral treatments become increasingly important. Effective management necessitates regular resistance monitoring and tailored treatment regimens in order to improve patient outcomes and prevent further resistance development.

## 3 SARS-CoV-2 drug resistance studies

### 3.1 Mutations in nsp12 (RdRp) leading to RDV resistance in SARS-CoV-2

Nsp12, situated within the palm subdomain, serves as a crucial component of the SARS-CoV-2 RNA polymerase, contributing to the active site necessary for RNA synthesis catalysis, a pivotal process in viral genome replication. Collaborating with other nonstructural proteins such as nsp7 and nsp8, nsp12 forms a functional RdRp complex, playing a central role in the assembly of the replication-transcription complex (RTC). Given its pivotal role in viral replication, nsp12 represents a prime target for antiviral drugs. Inhibiting the activity of RNA polymerase, particularly within the active site, can disrupt the viral life cycle and impede virus spread. Notably, mutations in Nsp12, particularly those occurring in the active site like the E802D mutation, can confer resistance to antiviral drugs such as remdesivir.

**Fig. 1:**
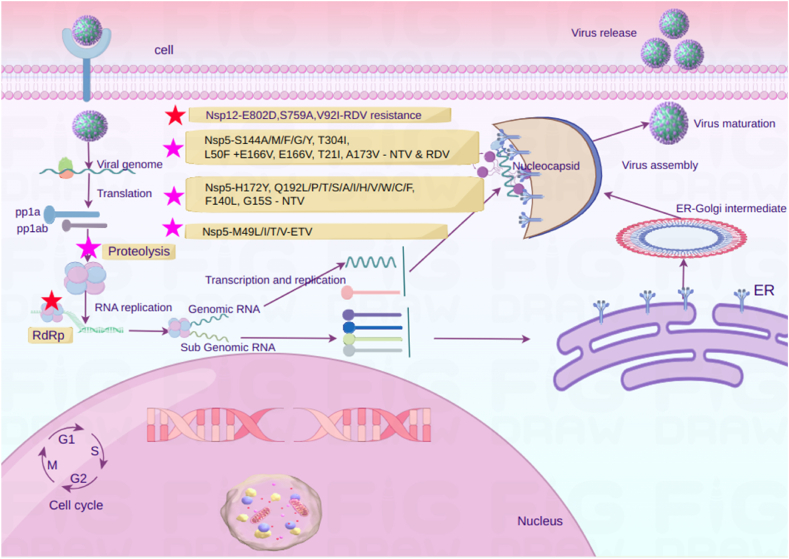
Mutations that cause drug resistance in the SARS-CoV-2. Key mutations in the RNA-dependent RNA polymerase (nsp12) include E802D, which increases resistance to remdesivir by approximately 2.5-fold. Other mutations like S759A and V792I also contribute to remdesivir resistance. In the main protease (nsp5), mutations such as E166V and S144A significantly reduce the effectiveness of protease inhibitors like nirmatrelvir. These mutations disrupt critical interactions necessary for drug binding, leading to increased resistance.

The first mutation identified in the RNA-dependent RNA polymerase (RdRp) nsp12, in an *in vitro* remdesivir resistance selection experiment, occurs at amino acid 802 [118], where glutamine is replaced by aspartate (Fig. 1) (Table 3). This mutation, located proximal to amino acids believed to interact with freshly synthesized RNA within the palm subdomains, results in approximately a 2.5-fold increase in IC50 for remdesivir [118]. The E802 residue within the palm subdomain plays a critical role in the active site of SARS-CoV-2 RNA polymerase, stabilizing the RNA-binding loop involved in nascent RNA binding through interactions with D804 and K807 in an electrostatic network [119]. According to the proposed mechanism by Szemiel and colleagues [118], the E820D mutation alters interactions between amino acid side chains, inducing minor structural changes in the affected region. This modification is thought to have an impact on nucleotide +3(nt+3) binding during template RNA synthesis, enabling elongation upon incorporation of an active form of remdesivir into the RNA [118].

**Table 3:** Reported SARS-CoV-2 mutations associated with drug resistance.

Mutation	Drug(s)	Drug target	References
E802D/A	Remdesivir	RdRp	[98,99]
S759A	Remdesivir	RdRp	[100]
V792I	Remdesivir	RdRp	[100]
C7995	Remdesivir	RdRp	[123]
V166L	Remdesivir	RdRp	[136]
L167F	Nirmatrelvir	3CLpro	[103]
H172Q/F	Nirmatrelvir	3CLpro	[104]
S144 A/M/F/G/Y	Nirmatrelvir, Ensitrelvir	3CLpro	[103,104,105,110]
H172Y	Nirmatrelvir	3CLpro	[104,105]
Q192 L/P/T/S/A/I/H/V/W/C/F	Nirmatrelvir	3CLpro	[104,105]
F140L	Nirmatrelvir	3CLpro	[103,105]
T304I	Nirmatrelvir, Ensitrelvir	3CLpro	[103,105,106]
L50F + E166V, E166V	Nirmatrelvir, Ensitrelvir	3CLpro	[103,105,106]
G15S	Nirmatrelvir	3CLpro	[105,109]
T21I	Nirmatrelvir, Ensitrelvir	3CLpro	[103,105,106,109,132]
A173V	Nirmatrelvir, Ensitrelvir	3CLpro	[103,110]
M49L/I/T/V	Ensitrelvir	3CLpro	[110]

A case report by Gandhi and colleagues in 2022 discussed the emergence of a resistance mutation to remdesivir in an immunocompromised patient with prolonged SARS-CoV-2 infection [119]. The study suggests that resistance to remdesivir, attributed to mutations at position 802 in nsp12, particularly affects immunocompromised patients, potentially rendering remdesivir treatment ineffective. The mutation at position 802 may modify the active site, either causing rejection of remdesivir or reducing steric clash induced by S861, thereby enabling the enzyme to avoid chain termination by remdesivir. The timing of the mutation's appearance in the patient, its specific location within nsp12, and its *in vitro* confirmation displaying resistance to remdesivir suggest a correlation between this mutation and subsequent elevation in viral shedding.

Stevens et al. investigated the development of remdesivir resistance, observing increases ranging from 2.7 to 10.4 times after multiple passages with progressively higher concentrations of GS-441524, the remdesivir precursor [120]. Genetic analysis revealed mutations in nsp12-RdRp, including S759A and V792I, with S759A located within the RdRp active motif. (Fig. 1) (Table 3). Introducing these mutations into a related coronavirus revealed transferability across betacoronaviruses, resulting in up to 38-fold RDV resistance and a replication defect. Biochemical analysis revealed that these mutations cause altered substrate preferences. Importantly, these resistance mutations were either rare or absent in sequences available to the public without RDV selection, emphasizing the need for continued research to understand the potential clinical emergence of such resistance.

Researchers at the US Centers for Disease Control and Prevention (CDC) investigated the possibility of other remdesivir resistance mutations [121]. Their study demonstrated a significant reduction in susceptibility to remdesivir with expanding Ebola virus in the context of rising remdesivir concentrations, identifying a common mutation, F548S, in the viral RNA-dependent RNA polymerase (RdRp) enzyme [121,122]. This mutation, previously associated with remdesivir resistance in SARS-CoV, underscores a shared mechanism of action against both viruses and highlights the importance of ongoing surveillance for such mutations in SARS-CoV-2 and Ebola to enhance understanding of potential drug resistance.

A case study reports on a patient with interstitial pneumonia who had previously undergone a lung transplant and then contracted COVID-19 from the Omicron BA.5 strain [123]. This patient demonstrated persistent viral shedding and fusogenicity. Following RDV treatment, genome-wide sequencing revealed multiple mutations, indicating dynamic changes in the viral population. The C799F mutation in nsp12 was especially significant because it conferred resistance to RDV by blocking RDV-triphosphate from entering the active site of the RNA polymerase.

Moreover, Cryo-EM analysis of SARS-CoV-2 RdRp bound to favipiravir revealed 72 interacting residues, from which 41 were chosen for mutation studies [124]. MOE (Molecular Operating Environment) simulations generated 350 single-point mutations, with 152 showing decreased affinity for favipiravir. A stringent cut-off identified 13 mutations, mainly in residues His439, Cys622, Asp623, and Thr680, as potential resistance hotspots. Further Rosetta-based design work confirmed that mutants with reduced affinity had higher RMSDs (root mean square fluctuation) and lower binding affinities compared to those with enhanced affinity. Sequence variation analysis emphasized that residues in the palm domain, especially those with significant diversity in affinity-attenuating designs, are crucial for understanding favipiravir resistance.

### 3.2 Mutations in nsp5 (3CLpro/Mpro) leading to NTV/ETV resistance

The coronavirus protease, also known as nsp5 or 3CLpro, plays a crucial role in processing viral polyproteins necessary for replication and is shared among all coronaviruses. Its structure and function are highly conserved. It is commonly referred to as the “main protease” (Mpro), as it is crucial for coronavirus gene expression. Its resemblance to the 3C proteases found in picornaviruses is further demonstrated by the term “3C-like protease” (3CLpro). Nsp5 proteases exhibit more than 80 % sequence identity among coronaviruses within the same genus, highlighting their similarity. As previously mentioned, 3CLpro represents a promising target for antiviral drugs, although ongoing evolution within SARS-CoV-2 raises concerns about drug resistance.

Iketani et al. investigate the resistance mechanism of specific mutations, focusing on their structural impact on antiviral drug binding, particularly Nirmatrelvir and Ensitrelvir [125]. Both drugs bind to the substrate-binding site but in distinct manners, leading to varied inhibition profiles among mutants.

The substitution of valine at position E166 weakens the three hydrogen bonds formed with Nirmatrelvir's lactam ring, resulting in significant drug resistance. E166, crucial for dimerization, faces interference with hydrogen-bonding interactions due to mutations at this site. Another pivotal residue for drug binding, S144, located within the S1 pocket and integral to the oxyanion hole alongside G143 and C145 [126], stabilizes the S1 subsite via hydrogen bonding. The S144A mutation disrupts this interaction (Table 3), affecting the binding of both Nirmatrelvir and Ensitrelvir. Four other mutants, namely S144M, S144F, S144G, and S144Y (Table 3), also exhibited enzyme activity comparable to the wild type (WT) among the top 15 frequently occurring mutations (Fig. 1) [126]. Notably, all five mutants demonstrated resistance to the drug nirmatrelvir, with their effectiveness reduced by 19.2–38.0 times compared to the normal version. While H172 does not directly interact with Nirmatrelvir, mutants H172Q and H172F exhibited enzyme activity comparable to the wild type (WT), displaying significant resistance to nirmatrelvir [126]. H172Y and H172A mutants demonstrated reduced enzyme activity but strong resistance to nirmatrelvir [126], and Pfizer also identified H172Y as a nirmatrelvir-resistant mutant [127] (Table 3). Additionally, Q192, positioned at the S4 pocket, engages hydrophobically with trifluoromethyl substitution in nirmatrelvir. The mutants Q192T, Q192S, Q192L, Q192A, Q192I, Q192P, Q192H, Q192V, Q192W, Q192C, and Q192F showed resistance to nirmatrelvir and enzyme activity similar to wild type (WT) [126] (Table 3). Mutation L167F, impacting the formation of the S4 subsite, leads to a steric clash with nirmatrelvir (Fig. 1) [125]. Similarly, the F140L mutation affects the interaction with both Nirmatrelvir and Ensitrelvir by breaking down the π-π stacking interactions between F140 and H163 [125].

According to a study by Zhou et al. (2022), nirmatrelvir experienced induced resistance through serial passages, resulting in substitutions such as T21I, T304I, L50F, and E166V (Fig. 1) (Table 3) [128]. Molecular simulations revealed that E166V and L50F + E166V reduced the binding of nirmatrelvir to Mpro, thereby diminishing the drug’s inhibitory potency and conferring resistance levels ranging from 40- to 175-fold [128]. Despite any associated fitness costs, some mutations facilitated by resistant variants showed high fitness and stability [128]. Preexisting substitutions, particularly L50F, were found to influence the selection of nirmatrelvir-resistant variations, potentially hastening resistance development [128].

Abdelnabi et al. evaluate the *in vivo* fitness of the nirmatrelvir-resistant virus in a hamster infection model, assessing the infectivity and transmission potential [129]. The evaluation of *in vivo* fitness in a hamster infection model demonstrates that the nirmatrelvir-resistant 3CLpro (L50F-E166A-L167F) virus effectively replicates in the lungs of Syrian hamsters [129]. The resistant virus-infected hamsters have lower viral RNA loads and infectious titers in their lungs than the WT virus-infected hamsters, despite their virus’s efficient replication [129]. Additionally, during co-housing experiments, the study suggests that direct contact between infected and non-infected hamsters may facilitate the transmission of the resistant virus [129].

In a recent case, an immunocompromised patient receiving B-cell depleting therapy (BCDT) for lymphoma exhibited severe SARS-CoV-2 infection symptoms despite s nirmatrelvir/ritonavir treatment [130]. Gene sequencing revealed de novo mutations (E166V and L50F) in the Mpro gene post-nirmatrelvir exposure, leading to increased viral replication and treatment failure [130]. This case highlights the clinical and virological indicators of nirmatrelvir treatment failure, marking the first instance of de novo Mpro mutations following nirmatrelvir exposure.

Ip et al. published a study after almost one year of nirmatrelvir/ritonavir approval [131], where they mentioned G15S as the most prevalent mutation present in several virus types (Table 3), which reduces the effectiveness of the nirmatrelvir. The second most prevalent mutation, T21I (Table 3), is found in some lineages in West Africa and South America, which also weakens the nirmatrelvir’s ability to stop the virus. A clinical report discusses a 55-year-old male patient with multiple myeloma, who developed a T21I mutation in the SARS-CoV-2 3CL protease after receiving nirmatrelvir therapy [132]. The patient initially responded to treatment, but then relapsed, with the resistant virus found in his nasopharyngeal swab. Despite experiencing temporary respiratory failure, he eventually recovered. Interestingly, the virus with the T21I mutation decreased during disease progression but increased during recovery while receiving remdesivir and dexamethasone. According to earlier research, E166V reduces viral fitness, but the L50F mutation can counteract this effect. L50F mutation was present in 7.7 % of the 13 sequences with E166V mutations. S144E is a different mutation that occurs in fewer than one sequence out of every million, but it showed the greatest reduction (470-fold) in nirmatrelvir's inhibitory activity on 3CLpro (Fig. 1).

A study conducted by Moghadasi et al. focuses on naturally occurring Mpro variants that show resistance to nirmatrelvir and Ensitrelvir [133]. Notably, the A173V mutation confers significant resistance to nirmatrelvir, whereas the M49L mutation confers strong resistance to ensitrelvir (Table 3). These mutations have occurred independently in various regions around the world, emphasizing the importance of tracking their prevalence. Research conducted on wild-type virus passages in the presence of ensitrelvir demonstrated that viruses with the M49L substitution were less susceptible to Ensitrelvir [134]. Furthermore, ensitrelvir treatment did not totally inhibit the growth of viruses with the M49L substitution *in vitro* or *in vivo*.

Structural analysis of the nirmatrelvir-Mpro crystal structure also identified 25 key interacting residues, resulting in 210 single-point mutants [135]. Ninety-four mutants showed increased resistance (positive dAffinity), with eleven exceeding a cut-off of >1.0 kcal/mol, mainly at positions 140, 143, 144, 165, 166, and 192. Positions 144 and 166 were crucial for resistance. About 40 % of predicted resistant mutations were already found in circulating SARS-CoV-2. High-frequency mutants like A191V, T190I, and S144L indicated resistance even without significant drug pressure.

Another case study involves a 65-years old immunocompromised patient with a history of malignant lymphoma who developed a resistant strain of SARS-CoV-2 despite extensive antiviral and antibody treatments [136]. Because of the loss of their natural B cells, the patient's immune response was weakened, contributing to the emergence of drug-resistant virus strains. By day 91, the virus had undergone several key mutations, including 3CLpro E166 A/V, which affects the efficacy of nirmatrelvir; S E340K, which affects sotrovimab; and RdRp V166L, which is associated with remdesivir.

Apart from the resistance mutations in Mpro that reduce the inhibitor binding, hyperactive mutations also play a key role in the evolution of resistance in circulating variants [137]. Hyperactive mutations are defined as the genetic changes that increase the activity of an enzyme. In a recent study using a FRET-based yeast assay, Flynn et al. discovered numerous hyperactive mutations that enhance Mpro activity [137]. These mutations are found both near and far from the active site, influencing protein stability and dynamics. Hyperactive mutations occur three times more frequently in lab-grown resistant viruses and circulating SARS-CoV-2 isolates than nirmatrelvir binding mutations. This suggests that hyperactive mutations play an important role in the natural evolution of drug resistance.

## 4 Therapeutic approaches for SARS-CoV-2

**Fig. 2:**
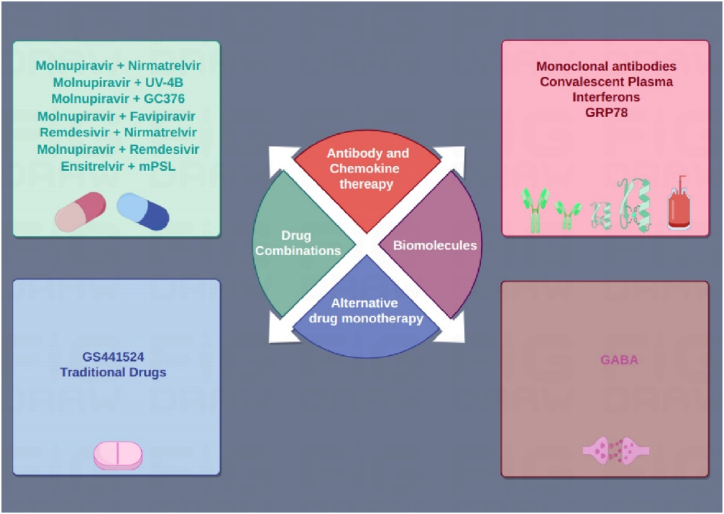
Therapeutic approaches to mitigate the SARS-CoV-2 drug resistance. These strategies include monoclonal antibodies, convalescent plasma, and interferons. Monoclonal antibodies, such as SA58 and TriSb92, show promise against Omicron variants when administered intranasally. Convalescent plasma therapy utilizes antibodies from recovered patients to treat or prevent severe COVID-19, particularly in immunocompromised individuals. Interferons, especially interferon-beta, are explored for their antiviral properties, with early administration recommended to prevent severe outcomes.

New therapeutic approaches for SARS-CoV-2 are essential due to the virus’s potential for mutability, which can impact the effectiveness of current treatments and vaccinations as well as create challenges with emerging variants (Fig. 2). Mitigating the risk of antiviral resistance, optimizing treatment efficacy, and easing the chronic symptoms of Long COVID are all critical. Addressing treatment gaps for breakthrough infections and individuals unable to receive vaccinations is also important. Furthermore, a wide variety of treatment alternatives ensures efficient management of the ongoing pandemic across various healthcare systems and advances global access and equity in healthcare. Investing in new treatments not only helps with the current crisis but also promotes improvements in research, technology, and treatment approaches, which in turn improves preparedness for pandemic in the future.

(i) Monoclonal Antibodies: As mentioned above, the specific monoclonal antibody treatment is restricted by the FDA due to concerns about its limited effectiveness against new Omicron variants. However, novel approaches, like the intranasal administration of antibodies like SA58 and trimeric “sherpa body” like TriSb92, offer potential advantages in safety and ease of use [138]. SA58 is a monoclonal antibody that is formulated as a spray. A Chinese study found significant efficacy in preventing symptomatic confirmed COVID-19 and lowering the likelihood of SARS-CoV-2 recovery from the nasopharynx, particularly against Omicron variants. This intranasal route of administration may offer benefits in terms of safety and ease of use [139]. TriSb92, a trimeric “sherpabody” is a small scaffold-targeting protein that has shown great potency against different SARS-CoV-2 variants in preclinical studies [140]. Monoclonal antibody research also explores the intricate interaction between neutralizing and non-neutralizing actions. For example, sotrovimab has been shown in animal studies to have strong binding, Fc-dependent effector cell functions, and the capacity to prevent infection, despite showing a decreased ability to inhibit some variants *in vitro* [141]. Diverse methods for assessing the efficacy of monoclonal antibodies are emerging as research advances. In addition to the conventional neutralizing capacity, non-neutralizing functions, and Fc effector interactions are being taken into account. The protective potential of monoclonal antibodies S309 and AZD7442 against Omicron strains has been investigated in recent *in vivo* efficacy studies in mice [142]. These studies have revealed varying mechanisms of action and underscored the necessity of ongoing surveillance of antibody countermeasure efficacy.

Despite challenges, these advancements in monoclonal antibody research are important steps forward in the ongoing fight against COVID-19.

(ii) Convalescent Plasma: Blood from recovered individuals is used in convalescent plasma therapy to produce antibodies that are used to treat illness. After removing blood cells, the remaining plasma which is rich in antibodies can help people recover from the same illness. Although not novel, this strategy was previously employed to treat diseases like Influenza and Ebola. In 2020, it gained prominence as a treatment for COVID-19 when no other treatment option was available. It is used to treat or prevent serious complications, particularly in cases where the immune system is slow to respond or when there’s no specific treatment. COVID-19 convalescent plasma is still available and is authorized for use in emergency situations especially for non-hospitalized individuals with compromised immune systems. Research on the effectiveness of COVID-19 convalescent plasma therapy is still ongoing. Fillipatos et al. (2023) recently conducted a meta-analysis [143], which included 34 randomized controlled trials, sought to determine the efficacy of convalescent plasma treatment in COVID-19 patients. In the end, convalescent plasma therapy was not found to be significantly associated with either improved secondary outcomes or a decrease in 28-day mortality in hospitalized populations. For outpatients, convalescent plasma therapy was linked to a 26 % lower risk of needing hospital care than standard care, which is a noteworthy exception. Regional variations in the efficacy of convalescent plasma may exist, as subgroup analyses in European trials revealed an 8 % lower risk of ICU-related disease progression. Even though its overall efficacy is unclear and has not been widely agreed upon, it remains a promising treatment option, especially when administered very early—ideally before COVID-19 symptoms appear.
(iii) Interferons: Interferons are proteins that are essential for the way the immune system responds to viral infections, such as COVID-19. The antiviral response of the immune system and the ability of interferon-alpha and interferon-beta to inhibit viral replication have been investigated as potential treatments for COVID-19. Clinical trials have looked into whether they should be used alone or in combination with other antiviral drugs. Although some studies point to positive aspects, research and discussion regarding the ideal patient populations, dosage, and timing for interferon therapy in COVID-19 are still ongoing.

Generally, interferon therapy has demonstrated positive effects in improving certain clinical aspects of COVID-19 and lowering mortality rates when used properly and in combination. Nevertheless, the efficacy of interferon therapy varies amongst research groups and may be influenced by variables like the disease stage or patient-specific genetic variations (polymorphism) [144]. On the other hand, the severity of the disease may worsen if interferons are administered later in the infection. The cause of this exacerbation is direct tissue damage and excessive inflammation, highlighting the significance of administration timing in COVID-19 interferon therapy [145].

Brzoska et al. (2023) proposed an approach for the successful interferon therapy of COVID-19 [146]. The proposed approach for using interferons in COVID-19 therapy advocates for the strategic selection of interferon type, favoring interferon beta over alpha due to its higher antiviral activity and lower association with autoantibodies in severe cases. Early administration, particularly through local applications such as inhalation or spray, is recommended within the first 3–5 days of symptoms onset to prevent severe outcomes in at-risk patients. Type III interferons are thought to be beneficial due to their anti-inflammatory properties, though their efficacy might be limited by receptor expression in specific cell types. It is advised to switch to daily systemic administration for later-stage treatments or if no improvement is seen by day 5. PEGylated formulations may be used in this situation. To reduce risks, more intensive therapy should not be started earlier than 6–8 days after symptoms first appear. After symptom days 10–12, administration should be carefully considered. This comprehensive approach aims to optimize COVID-19 therapy by balancing type-specific benefits, early intervention, and tailored dosages to improve efficacy and reduce potential side effects.

(iv) GABA receptor agonist: Gamma-aminobutyric acid (GABA), a major neurotransmitter system in the brain, and its receptors, or GABA-Rs, show protective effects against mortality and severe pneumonitis in infected mice. Pre-clinical research in K18-hACE2 mice infected with SARS-CoV02 suggests a promising role for GABA in COVID-19 therapy [147]. Against the odds, early GABA treatment limits neuroinflammation. Lowers lung viral loads and alters cytokine/chemokine levels linked to improved COVID-19 outcomes. GABA’s ability to reduce disease severity against various coronaviruses suggests that it could be used as a standard therapy for emerging SARS-COV-2 variants. GABA's human safety, room temperature stability, and availability as a dietary supplement make it an appealing candidate for clinical trials, indicating its potential as a cost-effective therapeutic strategy for COVID-19.
(v) Oral GS441524 derivatives: GS-441524 is a strong RNA-dependent RNA polymerase (RdRp) inhibitor with extensive antiviral activity. Due to its lower bioavailability, it is not quite successful against SARS-CoV-2. Remdesivir, the intravenous form of GS-441524, was the first SARS-COV-2 treatment approved by the FDA, but its efficacy remains unknown. Recent oral derivatives of GS441524, such as VV116, ATV006, and GS-621763, that target conserved viral RdRp are potential game-changers. Their oral administration aims to maximize clinical benefits by shortening the duration of COVID-19, reducing post-acute effects, and reducing side effects such as hepatic accumulation. ATV006, a mono-isobutyrate, shows improved efficacy against SARS-CoV-2 in preclinical studies [148,149], addressing the problem of poor oral bioavailability observed with GS-441524. However, since it’s still in the experimental stage, more research is needed to determine its tolerability, safety, and effectiveness. Tri-isobutyryl ester prodrug GS-621762, shows promise in lowering the SARS-CoV-2 burden in an animal model, highlighting its potential as a therapeutic option [150]. Considering these positive results, it is critical to recognize the paucity of clinical evidence for these derivatives, emphasizing the necessity for thorough investigations to confirm their efficacy and safety in the treatment of COVID-19.
(vi) Binding immunoglobulin protein (BiP): GRP78, also referred to as BiP, has drawn interest in regard to COVID-19 therapy due to its function as a host cell receptor for the SARS-CoV-2 virus' spike protein. Viral entry into cells is facilitated by this interaction, which aids in the infection process. Researchers are investigating the possibility of targeting GPR78 to disrupt its interaction with the virus, which could provide a therapeutic avenue. One potential way to slow down the spread of the virus is to create drugs or treatments that prevent the virus from binding to GPR78.

Binding Immunoglobulin Protein (BiP), previously thought to be an endoplasmic reticulum chaperone, has been discovered to be a multifunctional protein involved in cellular stress transduction across multiple compartments, including cell surfaces. The study by Rico-Llanos et al. demonstrates BiP as a promising therapeutic option for COVID-19 treatment [151]. The study highlights the relationship between inflammation and BiP-mediated cellular stress, especially in the context of COVID-19, where SARS-CoV-2 is able to identify BiP on the cell surface. Notably, BiP levels in blood or bronchoalveolar fluid are suggested as an early severity biomarker for the risk of pneumonia, providing a potent prognostic tool. With its capacity to reduce acute respiratory distress syndrome and regulate the inflammatory response, the anti-stress agent 4-PBA becomes a viable therapeutic option. The study reveals a lack of understanding regarding the cellular cascades that BiP regulates at the cell surface, and RIPK1 is suggested to be a possible mediator. This research establishes a link between stress and inflammation in ARDS, suggests that cell surface BiP exposure is a mechanism of severity, and introduces 4-PBA as a potential therapeutic option. It offers critical insights into reducing severe outcomes in COVID-19 and other inflammatory diseases.

(vii) Traditional/natural medicines: Investigating plant-based therapeutic strategies, such as traditional medicines and bioactive metabolites, has enormous potential to combat the COVID-19 pandemic. Recent studies into the possible use of herbs against COVID-19 have been prompted by prior research emphasizing the therapeutic benefits of herbs and their active compounds. These plant-based remedies have positive anti-COVID effects and are undergoing various stages of clinical trials. They can be used alone or in conjunction with conventional treatments. These herbal remedies can support the maintenance of the immune system, which can improve patients' overall well-being even though they might not be able to prevent viral infection. To effectively manage COVID-19 patients, it is imperative to thoroughly evaluate the safety and efficacy of phytochemicals and herbal remedies. In addition to using herbs, leading a healthy lifestyle, and taking dietary supplements can lessen COVID-19’s worldwide effects (Fig. 2).

Al-kuraishy et al. published a detailed review [152], encompassing all the traditional herbs and functional foods that could be used as a therapeutic option for COVID-19. Some of the medicines that are proven to be efficacious against COVID-19 are Lainhua qingwen [153], Qingfei toxic fuzheng [154], Shufang jiedu [155], Ayurvedic Kadha [156], tarpenes [71], Glycyrrhizin [157], polyphenols [158], alkaloids [159], Vitamin D [160], Vitamin C [161,162], Zinc [163], and Omega-3 [164, p. 3].

The protective effects of Asian traditional medicines, Withania somnifera (WS) and Tinospora cordifolia (TC), against COVID-19 were investigated in a study conducted by Rizvi et al. using hamster and hACE2.Tg mouse models [165]. The study found that WS, which is high in bioactive compounds, provided strong protection in hamsters by lowering body weight loss, lung viral load, and pulmonary pathologies. A reduction in pro-inflammatory cytokines and an increase in anti-inflammatory markers demonstrated the immunomodulatory potential of withanolides in WS. WS did not provide complete protection against severe COVID-19 in hACE2.Tg mice, though it did mitigate lung injury and modulate the adaptive immune response. Notably, both WS and TC had anti-oxidative properties, lowering the production of reactive oxygen species (ROS) and mitochondrial ROS (mtROS) in neutrophils. In particular, TC demonstrated a marked decrease in NETosis, a process of cell death linked to neutrophil extracellular traps. The results indicated that WS and TC may be able to reduce the severity of COVID-19 due to their combined antiviral and immunomodulatory mechanisms. This emphasizes the significance of investigating traditional herbal medicines in the context of infectious diseases.

## 5 Strategies to mitigate drug resistance

Mitigating drug resistance to COVID-19 involves a strategic combination of approaches.

### 5.1 Combination therapy as a potential strategy to reduce resistance

A potential strategy to slow the emergence of antiviral resistance is combination therapy, a multifaceted strategy that involves using several antiviral drugs at once (Table 4). By attacking the virus from several directions, this preventive strategy tries to limit its ability to adapt and become resistant to a particular drug (Fig. 2). It is much less likely for a viral population to acquire resistance mutations to all components at once when antiviral drugs with different mechanisms of action are used in combination. Combination therapy offers a more robust and long-lasting approach to address resistance and enhance overall patient outcomes, making it especially pertinent in the context of changing viral infections.

(i) Molnupiravir + Nirmatrelvir: Jeong et al. (2022) examined the therapeutic efficacy of antiviral drugs (NTV, RDV, MPV) and their combinations in SARS-CoV-2 infected mice [166]. In comparison to individual treatments or other combinations, the NTV-MPV combination showed superior antiviral activity, lower viral burden, and improved survival rates. The decreased efficacy of RDV was likely caused by enzymatic degradation. Both NTV and MPV individually and together have strong and long-lasting antiviral activity, which makes them attractive options for treating SARS-CoV-2. Very few scientists contributed to the research involving molnupiravir and nirmatrelvir combination therapy [167] and none of them mentioned whether it is effective against antiviral resistance or not.

**Table 4:** Combination therapies effective against SARS-CoV-2.

Drug Combinations	Target	Efficacy Evaluating Model	References
Molnupiravir + Nirmatrelvir	Viral RNA Replication	Preclinical, Clinical	[141,142,143]
Molnupiravir + UV-4B	Not specified	*In vitro*	[145]
Molnupiravir + GC376	Viral Protease Inhibition	*In vitro*	[144]
Molnupiravir + Favipiravir	Viral RNA Replication	In vivo (hamsters)	[146]
Remdesivir + Nirmatrelvir	Viral RNA Replication	*In vitro*, Clinical studies	[148,149,150,151,152]
Remdesivir + Molnupiravir	Viral RNA Synthesis	Pre-clinical, Clinical studies	[153,154]
Triple Combination	Multiple Targets (RNA Replication, Viral Entry)	Clinical trials	[147]
(RDV + NTV + mAB)			
Ensitrelvir + mPSL	Virus Entry Inhibition	In vivo (hamsters)	[155]

Recently, Marangoni et al. (2023) published a case report that discusses how a combination of two oral antivirals nirmatrelvir/ritonavir and molnupiravir was able to successfully treat a prolonged symptomatic SARS-CoV-2 infection in an immunosuppressed oncohematological patient [168]. Without causing any tolerability issues, the combination therapy produced rapid clinical and virological recovery. The authors stress that when evaluating antiviral compounds, it is crucial to rule out antagonism through pre-clinical research. According to the study [168,169], oral antivirals, as opposed to monoclonal antibodies, might still be effective against SARS-CoV-2 variants, and using combination therapy would reduce the likelihood of resistance.

(ii) Molnupiravir + UV-4B: Recently, Franco et al. published a study where they assessed the antiviral activity of the nucleoside polymerase inhibitor Molnupiravir (EIDD-1931) and the iminosugar antiviral UV-4B against SARS-CoV-2 variants (beta, delta, and omicron BA.2) in a human lung cell line [170]. All three variants were successfully suppressed by monotherapy with UV-4B and EIDD-1931 at physiologically possible concentrations. When compared to monotherapy, combination therapy with UV-4B and EIDD-1931 demonstrated a higher level of viral inhibition with additive or synergistic effects. The most strongly replicating variant, the delta variant, showed the highest EC50 values. According to the study, combination therapy may reduce viral burden more quickly, speeding up patient recovery and reducing transmission. Additionally, the combination proved effective in a model involving non-human primates. Nonetheless, the study recognizes the necessity of additional research *in vivo* and under dynamic drug concentrations.
(iii) Molnupiravir + GC376: Gidari et al. look into the combination treatment of molnupiravir with two proteinase inhibitors—nirmatrelvir and GC376, to treat SARS-CoV-2 [169]. Based on lessons from HIV and HCV therapies, the research emphasizes the critical role that combination therapy plays in the treatment of early-stage COVID-19 patients. In high-risk patients, molnupiravir has been shown to reduce hospitalizations and deaths by 30 %. Utilizing Calu-3 cell lines for SARS-CoV-2 *in vitro* testing, the molnupiravir combinations have additive or synergistic effects on the wild-type virus. Notwithstanding certain limitations, such as the utilization of non-human cell lines and the lack of specific virus variants, the research indicates a possible synergistic mechanism that impedes the SARS-CoV-2 exonuclease. Although statistical significance is not reached, testing on Omicron variants demonstrates a trend of viral titer reduction with the combinations. The findings highlight the necessity of additional research, particularly *in vivo* and human models, to confirm the effectiveness of these antiviral combinations in treating COVID-19 and getting ready for pandemics in the future.
(iv) Molnupiravir + Favipiravir: The synergistic potential of combining Molnupiravir and Favipiravir in treating SARS-CoV-2 infections has been clearly demonstrated in hamster models [171]. This combination has not only proven to be highly effective in lowering viral loads but also prevents the virus from spreading to uninfected contact sentinels. The combination demonstrated a strong antiviral effect even at suboptimal doses, with a median reduction of >4.5 log10 TCID50/mg lung tissue — beyond the predicted results from the administration of each drug alone. It is noteworthy that combination therapy is adaptive and works well even when started later than the infection. The observed spike in the overall number of mutations, specifically C-to-T mutations, in the viral RNA from combo-treated hamsters when compared to individual treatment groups is responsible for this success. The increased antiviral effect might be due to this higher mutation count, which could allow for the use of lower doses in clinical settings. This study using hamster models by Abdelnabi et a [171], shows that the combination of molnupiravir and favipiravir is a highly promising therapeutic approach. This suggests that clinical trials should be set up to assess the combination’s efficacy in treating COVID-19 humans.
(v) Triple Combination Therapy: Lately, Mikulska et al. published a comprehensive report [172], in which they investigate the characteristics and outcomes of a combination treatment plan for 22 severely immunocompromised patients with prolonged or relapsed SARS-CoV-2 infection. The novel approach uses two antivirals at the same time, mainly remdesivir and nirmatrelvir/r, and is often combined with monoclonal antibodies. The study demonstrates a significant efficacy rate, with a 75 % early virological response, a 73 % 30-day virological and clinical response, and an 82 % overall response rate when cases requiring a second course of treatment are considered. Notably, this is the largest cohort of immunocompromised patients ever subjected to such a combination treatment. The report highlights that patients with more severe infections are less likely to respond favorably and emphasizes the significance of mAbs in improving treatment outcomes. The study also shows that this combination therapy may be beneficial for people who have not had early antiviral treatment. The results offer significant insights into the promising efficacy and safety profile of combination therapy for critically immunocompromised patients dealing with prolonged or relapsed COVID-19, even though the study takes into account the limitations of small sample size and the lack of a control group.
(vi) Remdesivir + Nirmatrelvir: A study conducted by Pasquini et al. [173] discusses how immunocompromised people are still susceptible to severe COVID-19, especially those who have impaired humoral immunity due to conditions like B cell malignancies or B cell-targeting treatments. In a case series spanning three academic hospitals in Italy, the researchers treated 14 adult patients with persistent SARS-CoV-2 infection with a combination antiviral therapy consisting of remdesivir and nirmatrelvir/ritonavir. Most of the patients had previously received B cell-targeting therapies and had been diagnosed with B cell lymphoma. The combination therapy, which lasted a median of 10 days, resolved COVID-19 symptoms in a median of 6 days and cleared the virus in a median of 9 days, despite differences in the severity of the disease. These findings highlight the need for more research through prospective comparative trials and suggest that the remdesivir and nirmatrelvir/ritonavir combination may be effective in treating persistent COVID-19 in immunocompromised patients with compromised humoral immunity. Before this study, the synergy between remdesivir and nirmatrelvir/ritonavir is also reported by several researchers in *in vitro* and clinical studies [174,175,176,177].
(vii) Molnupiravir + Remdesivir: In order to treat patients with severe COVID-19, Hashemian et al. looked into the possible synergistic effects of combining molnupiravir with remdesivir [178]. The purpose of this study was to evaluate the effects of combining these two antivirals in five cases of severe COVID-19. A thorough assessment of data was, however, hampered by a number of limitations, such as a small sample size, the lack of a control group, and ethical considerations while combining molnupiravir with remdesivir. Although the combination might have a synergistic effect, specific results are still unknown, highlighting the need for more research to determine the therapeutic efficacy and possible risks of this combined antiviral approach, especially in severe and hospitalized cases.

Previously, Abdlenabi et al. [179] also investigated the effectiveness of the combination therapy involving molnupiravir and the parent analog of remdesivir (GS-441524). Both *in vitro* and in infected hamsters, the combination showed a strong antiviral effect against the virus, especially against the beta (B.1.351) variant. In cell cultures, the researchers observed a general additive antiviral effect with noticeable synergism at specific concentrations. Furthermore, the combination of GS-441524 and molnupiravir demonstrated a potent antiviral effect in hamsters given suboptimal doses of each drug, lowering viral titers in the lungs to undetectable levels in the majority of the animals.

(viii) Ensitrelvir + mPSL (methylprednisolone): Most recently, Sasaki et al. conducted a study that focuses on the effects of antiviral and anti-inflammatory treatments on SARS-CoV-2 infection in hamsters [180], to determine their efficacy in reducing the severity of COVID-19. SARS-CoV-2 targets lung bronchial and alveolar epithelial cells, causing inflammation and immune cell infiltration. According to the study, antiviral therapy administered early is essential for preventing hyperinflammation and lessening the severity of the illness. The study highlights that more severe outcomes result from delayed intervention by comparing the effects of treatment with ETV and mPSL. Surprisingly, combination therapy combining ETV and mPSL is suggested as a powerful treatment choice that efficiently regulates host inflammatory reactions without postponing viral elimination. The study does, however, acknowledge certain limitations, including the hamster models and the requirement for additional research on drug interactions and different oral antiviral candidates.

Although several efficacious combination therapies have been identified, none of them have been proven beyond a reasonable doubt to address drug resistance in the context of SARS-CoV-2. Drug resistance in the fight against SARS-CoV-2 remains an unresolved issue despite continued research and the identification of promising therapeutic combinations. This emphasizes the necessity of ongoing research and the development of innovative approaches to address the problems with drug resistance caused by the virus.

### 5.2 Rational drug design or drug repurposing

Rational drug design and drug repurposing are two prominent approaches to mitigate drug resistance to COVID-19. An in-depth knowledge of the molecular and biological characteristics of the virus is necessary for rational drug design, as it allows researchers to identify the precise targets that are essential for the virus's replication or the host's reaction. Using this knowledge, novel therapeutic agents with high precision can be designed, aiming to interact selectively with these identified targets. This technique has the advantage of tailoring drugs for maximum potency while minimizing the risk of side effects. Furthermore, since the drugs aim to disrupt the critical stages of the viral life cycle, the targeted nature of rational drug design can reduce the likelihood of the virus developing resistance.

Drug repurposing, on the other hand, entails the investigation of existing drugs that were originally developed for various health conditions. This strategy takes advantage of these drugs' known safety profiles and mechanisms of action, screening them to identify candidates with potential efficacy against COVID-19. The potential for faster deployment is what makes drug repurposing appealing since many of these drugs have already undergone extensive testing for safety and regulatory approval. Repurposed drugs may also have a wider range of antiviral activity since they can act on several targets. This diversity can be especially helpful in addressing the virus's complexity and reducing the likelihood that resistance will develop.

One major example of a repurposed drug for SARS-CoV-2 is Remdesivir, originally evaluated for the Ebola virus in 2014. It has improved clinical outcomes and decreased mortality risk in both hospitalized and non-hospitalized patients [37]. Zidovudine (AZT) has the potential to inhibit SARS-CoV-2 polymerase, as demonstrated in silico with stable interactions [181]. Lamivudine, another HIV drug, showed antiviral activity against SARS-CoV-2 *in vitro* [182] and reduced infection rates in Hepatitis B patients, making it a promising candidate for COVID-19 treatment [183]. Abacavir also shows promise due to its ability to inhibit viral polymerase [184]. Additionally, tenofovir and emtricitabine have been found to reduce viral titers in non-hospitalized patients [185]. Despite the initial promise, many other drugs were later revoked by the FDA due to failure in clinical trials or toxicity concerns.

However, repurposing a drug for a new use involves many challenges, which often leads to a high rate of failure. These challenges include proving a strong pharmacokinetic/pharmacodynamic (PK/PD) relationship, confirming a safe profile for the new indication, determining an appropriate potency, maintaining target engagement, and distinguishing the repurposed drug from the standard of care. Repurpose drugs also carry a commercial risk because it can be challenging to obtain new patents and exclusive market rights for the drug’s new use [186]. Sometimes, the original drug developers discover new uses for a drug in preliminary research or as a side effect in clinical trials, and then they patent these new uses. A well-known example is sildenafil, which was actually developed to treat high blood pressure but later became Viagra when it was found to be quite effective for treating erectile dysfunction during clinical trials [187]. To increase the chances of successfully repurposing and approving a drug, all of the challenges must be carefully considered.

Bringing together the benefits of both drug repurposing and rational drug design is a holistic and synergistic approach. Rational drug design provides accuracy and adaptability by finding novel targets or improving the effectiveness of already-approved drugs. Simultaneously, drug repurposing leverages the abundance of knowledge surrounding currently available drugs to enable the quick identification of viable solutions.

### 5.3 Continuous monitoring of viral mutations

In the context of COVID-19, regular monitoring of viral mutations is a critical approach for reducing drug resistance. Scientists can quickly detect new variants and evaluate how they might affect the effectiveness of treatments by using systematic genomic surveillance. This enables the early adjustment of antiviral drug regimens and vaccination plans. This proactive strategy emphasizes the value of data sharing for thorough surveillance by bringing together scientists, public health organizations, and policymakers globally. As specific mutations are discovered, researchers can investigate whether existing vaccines are still effective or if improvements are required, contributing to a dynamic and adaptive response. With over 2 million SARS-CoV-2 genomes sequenced and shared via the GISAID platform, the COVID-19 pandemic has signaled a significant advancement in the application of whole-genome sequencing (WGS). This huge genomic data has been critical in informing public health decisions, such as detecting mutations that may affect the virus's behavior, such as virulence, spread, and ability to evade the immune system. While genomic sequencing offers quick insights, it is still difficult to predict how these genetic alterations will impact the virus's properties.

Ongoing genomic surveillance informs public health policies, enabling policymakers to adjust strategies in response to new variants with higher transmissibility or immune escape. Integrating genomic data with clinical insights enhances our ability to preemptively address challenges posed by evolving viral genetics. A notable example is the Traveler-Based SARS-CoV-2 Genomic Surveillance Program launched by the CDC in September 2021. Initially deployed at three U.S. airports, this program is set to expand to eight international airports by spring 2024, focusing on identifying new variants from travelers. Similarly, the United States' National SARS-CoV-2 Strain Surveillance (NS3) system demonstrates practical genomic surveillance. NS3 collects and analyzes data on SARS-CoV-2 genetic diversity nationwide. By sampling from various regions, it tracks virus evolution, identifies emerging variants, and evaluates their impact on diagnostics, treatments, and vaccines. This system supports timely public health decisions and adaptive strategies. Wastewater surveillance is another key method, involving the monitoring of wastewater for SARS-CoV-2 traces. This technique offers early warnings of new variants and potential outbreaks by analyzing genetic material at a community level, complementing traditional surveillance methods.

The integration of genomic surveillance with national programs and innovative methods like wastewater analysis exemplifies a comprehensive approach to managing viral mutations. Coordinated global efforts enhance our ability to adapt public health measures and respond effectively to emerging variants of SARS-CoV-2 and other infectious threats. This strategy is crucial for both current pandemic management and future preparedness.

### 5.4 Public health interventions to reduce transmission

Drug resistance in SARS-CoV-2 requires a multifaceted approach centered on public health interventions. Aiming for broad coverage, vaccination campaigns have the dual benefit of directly preventing infections and acting as an effective barrier to the excessive use of antiviral drugs. These campaigns effectively reduce the selective pressure that could lead to the development of drug-resistant strains by reducing reliance on specific classes of drugs. Besides vaccinations, reliable testing and early detection are essential components of the strategy as they enable immediate isolation and treatment, which limit the uncontrolled spread of the virus and minimize the need for a lot of antiviral drugs. Efficient contact tracing augments these efforts by identifying and isolating potentially exposed individuals, thereby breaking the transmission chain. Campaigns for public awareness help the cause by informing the public about preventative measures, which in turn lowers the rate of transmission. To reduce the risk of resistance emergence, antiviral stewardship programs in healthcare settings monitor medication use, encouraging cautious deployment and minimizing needless prescriptions. International cooperation, the exchange of knowledge about new variations, and continuous research into novel antiviral medications expand the range of available treatments and taken together, provide a comprehensive approach that is essential for reducing SARS-CoV-2 drug resistance. These coordinated efforts tackle the long-term objective of reducing the risks of resistance emergence alongside the immediate pandemic challenges.

## 6 Future directions in SARS-CoV-2 drug development

Antiviral drug discovery has advanced significantly, especially with the pressing need to combat SARS-CoV-2. Before the COVID-19 pandemic, the main focus in antiviral drug development was on treating HIV and hepatitis C. These two viruses were the target of over 67 % of the antiviral drugs that had been approved. Developing new antiviral drugs typically took a very long time, often stretching over several decades, particularly for the initial treatments designed to combat a new virus. However, after the COVID-10 pandemic, multiple antiviral treatments were identified, tested, and approved in less than two years. This was achieved by applying previous knowledge and quickly adapting to the urgent situation. Key to this success were enthusiastic teams and thorough planning, which included having backup strategies. The large investments needed to accelerate the process highlighted the challenge of adapting traditional, cautious public funding to support fast-paced drug development. The pandemic also saw remarkable collaboration among drug companies and government agencies, leading to new partnerships and networks like ACTIV8 and RECOVERY, which significantly sped up clinical trials [188]. Regulatory agencies responded swiftly by implementing faster review processes, though they had to continually adjust trial criteria as the pandemic evolved. For future pandemics, it will be essential to develop tools and drugs for potential new viruses and invest in drug discovery infrastructure. This will help ensure a more effective and rapid response to global health crises.

Disrupting the viral replication mechanism is a major area of focus, and RNA-dependent RNA polymerase (RdRp) has emerged as a key target. Remdesivir and other drugs that inhibit RdRp have been helpful in the treatment of COVID-19. Researchers are also investigating immune response modulation and host cell factors to impede the virus’s life cycle and strengthen the body’s defenses. Through drug repurposing strategies, the development of broad-spectrum antivirals capable of combating multiple viruses is gaining attention. Protease inhibitors are being researched for their potential to treat SARS-CoV-2, as they have been successful in treating other viral infections. To improve treatment outcomes and lessen the severity of infection, the interplay between antiviral drugs and vaccines is becoming increasingly important. Likewise, by predicting efficacy and optimizing chemical structures, the combination of artificial intelligence and computational approaches speeds up the drug discovery process. In order to build on existing knowledge and to quickly address new challenges, researchers must now collaborate globally and share data. To stay ahead of the changing viral landscape, SARS-CoV-2 drug development is likely to take a multifaceted approach in the future.

A multifaceted approach to combating viral pathogens significantly enhances effectiveness by targeting viruses through various strategies simultaneously. For instance, combining vaccinations, antiviral drugs, and public health measures creates a robust defense system that tackles the virus at different stages, reducing overall infection rates and severity. This approach also helps in minimizing the development of resistance, as viruses struggle to adapt to multiple concurrent interventions. Public health responses become more effective, speeding up containment and recovery, as evidenced by the coordinated efforts during the COVID-19 pandemic, which combined vaccines, testing, and social distancing. Additionally, this strategy fosters greater public engagement and compliance, leading to improved health outcomes and quality of life, as seen with chronic infections like hepatitis C, where a combination of treatments and lifestyle adjustments yields higher cure rates and better disease management.

Drug development encounters numerous challenges throughout the process. Finding promising drug targets and developing effective compounds in the early stages is difficult and demands extensive research and optimization. This stage is critical and uncertain because preclinical testing in animal models frequently does not perfectly predict human outcomes. Clinical trials pose additional challenges, such as finding appropriate volunteers, high costs, and requiring thorough evaluations of safety and effectiveness. Clinical trials present additional challenges, such as recruiting suitable participants, high costs, and rigorous safety and efficacy assessments. Navigating the regulatory landscape to obtain approval from agencies such as the FDA or EMA adds another layer of complexity, requiring meticulous documentation and adherence to strict guidelines. All things considered, numerous scientific, financial, and regulatory roadblocks can obstruct advancement and drive up costs during the discovery to market phase.

The integration of novel technologies like gene editing and RNA-based therapies indicates a promising future for SARS-CoV-2 drug development. CRISPR-Cas9 gene editing tools enable the modification of host cell factors exploited by the virus, making cells less susceptible to infection. This strategy produces genetically altered cells that are resistant to SARS-CoV-2, potentially providing a long-term defense. A colorimetric viral detection method using the CRISPR/dCas9 system, developed by Jeong Moon et al. [189], detects RNA from viruses through a color change reaction. This CRISPR/dCas9-based method is particularly valuable for identifying SARS-COV-2 and its drug-resistant variants due to its rapid and simple detection capabilities. Using the CRISPR/dCas9 system, it can quickly identify specific RNA sequences associated with the virus, including drug resistance mutations, through a simple color change reaction. Sierra SARS-CoV-2, an open-source, web-based tool introduced by Tzou et al. [190], can also be employed to detect drug resistance in SARS-CoV-2 by analyzing viral sequences for specific mutations associated with reduced susceptibility to antiviral. It utilizes global mutation prevalence data and the CoV-RDB database to identify mutations that might indicate resistance to treatments like antiviral drugs or monoclonal antibodies. Sierra SARS-CoV-2 analyzes spike and other viral protein mutations, to track and understand resistance patterns, aiding in the monitoring and management of emerging drug-resistant SARS-CoV-2 variants. Other bioinformatic tools, such as SABRes [191], are designed to detect drug resistance mutations in SARS-CoV-2 genomes and viral subpopulations by utilizing expanding public datasets. Recombinase Polymerase Amplification (RPA) is an isothermal nucleic acid amplification method that operates at a constant, low temperature (37–42 °C), allowing for rapid and simple detection of specific DNA or RNA sequences without the need for thermal cycling. In the context of SARS-CoV-2, RPA can be employed to rapidly identify mutations associated with drug resistance, allowing for timely monitoring and management of resistant strains in various testing scenarios. Dried blood spot (DBS) testing, which has previously been shown to be effective in detecting drug resistance in HIV-1 and HIV-2 [192,193] has potential applications in the monitoring of SARS-CoV-2 drug resistance. Similar to its use in HIV, DBS testing can be employed to analyze SARS-CoV-2 for resistance mutations by assessing viral genetic material from blood samples. Additionally, DBS testing is utilized for monitoring SARS-CoV-2 antibody levels [194], demonstrating its versatility and applicability in the field of viral infections.

Another approach for SARS-CoV-2 drug development is RNA interference (RNAi), in which small RNAs can be designed to target viral RNA, inhibiting translation, and disrupting viral replication. Pfizer-BioNTech and Moderna's success with mRNA vaccines exemplifies the potential of mRNA-based therapies beyond vaccination. Therapeutic proteins that directly impede viral replication or alter the host immune response can be encoded by mRNA, enabling a prompt response to newly emerging viral variants. Advances in gene editing, such as base editing and prime editing, provide accurate methods for modifying DNA, facilitating the creation of treatments that more specifically target the viral genome or host factors. The flexibility of these technologies offers a robust and dynamic approach to drug development, able to tackle novel challenges brought about by emerging viral strains, even as SARS-CoV-2 continues to evolve.

Proactively combating potential resistance to antiviral therapies is a critical consideration in the ongoing pursuit of effective drug development for SARS-CoV-2. In the future, combination therapies will play a major role in combating resistance through a multifaceted approach. Resistance to a comprehensive treatment regimen is more difficult to develop when multiple drugs targeting different viral mechanisms are combined. Simultaneously, scientists are investigating the idea of multi-target antiviral drugs, which aim to simultaneously disrupt multiple viral proteins or host factors, thereby raising the barrier at which the virus can evolve resistance. Another cutting-edge approach is host-targeted therapy, which focuses on altering important host cell components for viral replication. Rather than going after viral components directly, this strategy uses the host's cellular machinery to reduce the likelihood of resistance while also broadening the antiviral strategy. Adaptive therapies, which involve real-time monitoring of viral genomic changes and subsequent adjustments to treatment regimens, are also gaining popularity. This dynamic strategy enables a rapid response to emerging viral variants, ensuring that antiviral interventions remain effective. These approaches, taken together, highlight a comprehensive and forward-thinking approach to fighting resistance in the evolving landscape of SARS-CoV-2 drug development.

## 7 Conclusion

Although COVID-19 is no longer considered a global emergency, ongoing research remains important, particularly for combating drug resistance in immunocompromised and high-risk populations. To date, no combination therapies have been proven effective in overcoming SARS-CoV-2 resistance; however, history shows that such strategies can be highly effective, as evidenced by treatments for viruses like influenza and HIV. This emphasizes the urgent need to investigate the mechanisms underlying drug resistance and the virus's ongoing evolution.

By analyzing the complex interactions between the virus and its host and exploring potential therapeutic interventions, we pave the way for a more nuanced and adaptable approach to managing drug resistance. Adopting a multifaceted approach, including combination therapies, targeted drug development, and continuous surveillance of viral mutations, is essential in this fight. Despite the challenges ahead, ongoing collaboration among scientists, the pharmaceutical industry, and global health organizations is critical.

This paper serves as a call to action for continued research, heightened awareness, and collective efforts to develop robust strategies to mitigate the impact of drug-resistant strains and protect the health of global populations as we confront the ongoing challenges posed by SARS-CoV-2.

## CRediT authorship contribution statement

**Sania Batool:** Writing – original draft, Conceptualization. **Santosh Chokkakula:** Visualization. **Ju Hwan Jeong:** Writing – review & editing. **Yun Hee Baek:** Writing – review & editing, Funding acquisition. **Min-Suk Song:** Writing – review & editing, Funding acquisition, Conceptualization.

## Ethics declaration

Review and/or approval by an ethics committee was not needed for this study because this is a scoping review using existing publications without any primary or secondary data collection.

## Statement

During the preparation of this work the authors used ChatGPT 3.5 in order to scientifically edit the manuscript to improve readability and language, and to identify and rectify grammatical errors. After using this tool, the authors reviewed and edited the content as needed and take full responsibility for the content of the publication.

## Data availability Statement

There is no data for this review.

## Funding

This work was supported by the 10.13039/501100003653Korea National Institute of Health, the 10.13039/100018688Korea Disease Control and Prevention Agency (6634-325-320-01 to M-.S.S.), and the 10.13039/501100003725National Research Foundation of Korea (NRF- 2021R1A2C2006961 to M-.S.S), and the Korea Health Technology R&D Project through the 10.13039/501100003710Korea Health Industry Development Institute (KHIDI), funded by the Ministry of Health & Welfare, Republic of Korea (grant number: HI23C0392 to Y.H.B).

## Declaration of Competing Interest

The authors declare that they have no known competing financial interests or personal relationships that could have appeared to influence the work reported in this paper.
